# Biobanks and scientists: supply and demand

**DOI:** 10.1186/s12967-018-1505-8

**Published:** 2018-05-22

**Authors:** Angelo Virgilio Paradiso, Maria Grazia Daidone, Vincenzo Canzonieri, Alfredo Zito

**Affiliations:** 1 Oncologia Medica Indirizzo Sperimentale & Direzione Biobanca, Istituto Tumori G Paolo II, Istituto di Ricovero e Cura a Carattere Scientifico (IRCCS), Bari, Italy; 2 Department Experimental Oncology and Molecular Medicine, Fondazione IRCCS Istituto NazionaleTumori, Milan, Italy; 3 Pathology Unit and Biobank, Centro Riferimento Oncologico (CRO), IRCCS, National Cancer Institute, Aviano, Italy; 4 Pathology Unit, Istituto Tumori G Paolo II, IRCCS, Bari, Italy

**Keywords:** Biobank, Biospecimen, Translational medicine, Underuse

## Abstract

The biobanks, providers of biospecimens, and the scientists, users of biological material, are both strategic actors in translational medicine but the communication about those two subjects seems to be delicate. Recently, biobank managers from US and Europe stressed the danger of underuse of biospecimens stored in their biobanks thus stimulating the debate about innovative ways to collect samples and to communicate their availability. We hypothesize that the already stored collections meet the interest of present scientists only in specific situations. Serial biospecimens from patients with large associated clinical data concerning voluptuary habits, environmental exposure, anthropomorphic information are needed to meet the even more specific projects the scientists are planning. The hypothesis of activation of specific sections in ranked journals aimed to facilitate the communication between partners interested in finding/collecting ad hoc biospecimens is discussed.

Human research biobanks can be defined as structured collection, specifically developed as resources for research, of high-quality human biological materials associated with clinical/biological data and potentially organized to facilitate biospecimens sharing among scientists [1].

Human biobanks have been variously classified (according to donor characteristics, design of the accrual, type of biospecimens collected, conditions of storage, sponsorship, etc.) [2] but a simple classification referring to type of research intended to be supported (population study, basic research, translational study, clinical trial) has been also suggested [3].

Recently, the recent development of innovative technological tools and the need for personalized approach to each patient, has led to the development of research biobank structures where biospecimens collection, storage conditions, data handling are managed according to high quality standards. A primary emerging need from this situation is the development of a real biobank science in which knowledge of ethical-legal local regulations, and expertise of biobank operators through specific education/training program play a major role. Sometimes, the biobanks have been planned as population-wide collection, as large inter-institutional specimen centralized-collections (i.e. ORIEN biobank, led by Ohio state and several US universities), or, even, as world-wide initiatives (i.e. Wellcome Trust Sanger Institute, Cancer Genome Project). An updated inventory data about the bioresources, describing availability of various resource types such as biological material, data, expertise, and offered services in Europe has been recently published [4].

The relevance that such structures have in a modern research scenario is demonstrated by the constant increase in biobank number. A recent US survey reported that two-thirds of operating biobanks have been established within the last decade [5].

However, the debate about the sustainability of human research biobanks and use of preserved biospecimens is heated. The costs of requested technologies [6], the strategic role of biospecimens in translational research [7] and ethical issues related to the responsibility to preserve and utilize human tissues for public benefit [8], contribute to become the discussion stimulating from several points of view and crucial for the development of personalized medicine.

A critical point stressed by several recent reports is the underuse of the biological material preserved in biobanks. Henderson [9] reported that “many biobankers are worried about underutilization of specimens”. Scudellari [10] stressed that this is not a problem unique to US institutions.

Several issues have been identified as limiting steps for the effective utilization of biobanked specimens: the low quality [11] but also the unknown, undocumented or uncertifiable quality [12] of the collections have been described as a main problem by several biobankers; the presence of an efficient model of governance supported by an optimal workflow and informatics, chain of custody, centralized Institutional Review Board (IRB), unrestricted policies have been also considered essential to facilitate and encourage the involvement of biobanks in translational research [13]; a lack of proper advertise of available collections at institutional and external conferences is retained to be a further relevant negative factor [9]; we already stressed the problem of limited involvement of patients and civil society in direct governance of biobanks [14]. Puchois [15] debated the relevance of access policies, sometime explicitly excluding investigators who are associated with drug-biomedical tools for profit companies from the access to biospecimens. Lastly, a sort of academic prudishness limiting the distribution of samples from academy to industry has to be mentioned; what a shame, since a majority of new diagnostics and drugs come from industry.

More specific comments concern peculiar characteristics of the collections.

A continuous analysis of research trends [16] and innovative biotech approaches [17] are necessary in order to meet the evolving biospecimen needs for groundbreaking aspects of the scientific world. This problem refers to two aspects: the availability of associated new and relevant clinical biological data to preserved biospecimens; the adoption of appropriate sampling procedures for biospecimens to be biobanked for new trial designs.

The research in cancer is even more frequently conducted in series of biospecimens from cohort of subjects with very specific habits, clinical, pathological, biomolecular, environmental characteristics [18] and only occasionally series of biospecimens with these features are available or at least advertised as available by biobanks. Some biobanks are organized to acquire basal and updated information of participants to specific cohorts of subjects such as disease follow-up, voluptuary habits, work conditions, thus laying the foundations for highly specialized environmental researches also. Furthermore, biomarker and genetic data belonging to biobanked samples may have to return to the Biobank to continuously enrich the research value of the single biospecimen and to permit its utilization in further more detailed studies. These considerations should motivate biobanks to move from the concept of “minimum essential data” [19] to the hypothesis of a wide “maximum” accompanying kit of data associated with each sample.

An example of such approach is represented by our collection, at Institute Tumori-Bari, of blood samples from 250 healthy heavy smokers followed for more than 5 years. Information on familiarity, anthropometric characteristics and all voluptuary habits have been associated to each biospecimen. These samples could represent an interesting mine for scientists involved in lung cancerogenesis, exposure to xenobiotics, biological damage and so on.

New trials focused their attention on availability of patient’s biospecimens fitting for innovative lab approaches. An eloquent example is represented by sampling multiple synchronous biospecimens (normal tissue, pathological tissue, blood, urines, etc.) from the same patient useful to individualize germinal and/or somatic genetic characteristics of pathogenic relevance for several cancers. The availability in our biobank of a series of 500 women with 5 years of follow-up, provided of a complete set of familial and clinical data, with presurgical blood and surgically removed tissues from normal and tumor breast could represent the basis for new scientific ideas.

A last example of such approach moving towards a modern biobanking activity could be represented by the innovative design of N-of-1 trial [20] in which serial sampling of liquid biopsies are requested to monitor the biomolecular progression of the cancer. Are there biobanks organized to routinely collect biospecimens from blood serial samples?

Those are only examples of how biobankers and scientists should more deeply and urgently interact to optimize the support to bench research. If we agree about such a view, we should think about new ways to facilitate communications about those main actors: academic scientists, industry scientists and biobankers. Ranked journal should provide a specific section in which accreditated biobanks could have the possibility to describe peculiar collections of biospecimens they possess and, conversely, scientists could find the possibility to describe the characteristics of ad hoc series of samples they are looking for these information could concern collected samples already stored or series of samples to be prospectively collected. An example of such approach at the individual sample level, is http://www.ispecimen.com, a functional platform that could really enhance sample utilization through personalized sample request.

Allen [21] suggests that “the goal is to form small, flexible, ad hoc groups of biobanks for each sample request, based on the type of sample and the difficulty of collection”.

In this framework, why do not think to a “personalized” request approach by the researchers to an Institutional Biobank? The experience of Bio-banking “on demand”, firstly developed at the CRO Aviano Cancer Center, is moving towards this direction. Scientists can ask for biomaterials not routinely collected (e.g. sebum). biomaterials collected in additional timings (e.g. after n cycles of therapy); samples collected from the general population (e.g. patients with negative colonoscopy to be used as “real” negative control of colon cancer patients). Preliminary results coming from this “on demand” approach seem positive and directly influencing the satisfaction of the researchers and, not for last, the awareness of the participants/patients donating their biospecimens on their valuable contribution.

This is what usually happens in the even faster world of work: supply and demand counteract, dynamically and quickly.

## Abbreviations

IRCCS: Istituto di Ricovero e Cura A Carattere Scientifico
CRO: Centro Riferimento Oncologico
